# Persistent Neonatal Hypocalcemia as an Early Clue to TCIRG1-Related Infantile Osteopetrosis in an Infant of a Diabetic Mother: A Case Report

**DOI:** 10.7759/cureus.115229

**Published:** 2026-08-26

**Authors:** Sahar D Alshehri, Fuad M Alkudaysi, Turki Alhasani, Ali Alghanmi, Mohammed A Albariqi

**Affiliations:** 1 Pediatric Endocrinology, King Faisal Medical City, Southern Region, Jeddah, SAU; 2 Pediatrics, South Al-Qunfudah Hospital, Al-Qunfudah, SAU; 3 Pediatrics, Khamis Mushayt Maternity and Children Hospital, Khamis Mushayt, SAU; 4 Medicine and Surgery, Umm Al-Qura University, Al-Qunfudah, SAU

**Keywords:** diagnostic challenge, hematopoietic stem cell transplantation, infantile malignant osteopetrosis, infant of diabetic mother, neonatal hypocalcemia, osteosclerosis, tcirg1

## Abstract

Infantile malignant osteopetrosis is a rare autosomal recessive skeletal disorder caused by impaired osteoclast-mediated bone resorption, resulting in diffuse osteosclerosis, bone marrow failure, neurologic complications, and metabolic disturbances. Early diagnosis remains challenging, particularly when initial manifestations overlap with common neonatal conditions. Persistent neonatal hypocalcemia may be an early but underrecognized clue. We report a Saudi male infant with genetically confirmed TCIRG1-associated osteopetrosis who presented with persistent neonatal hypocalcemia and secondary hyperparathyroidism. Born at 36 weeks to a mother with insulin-dependent diabetes, he required neonatal intensive care for respiratory distress, hypoglycemia, and a right clavicular fracture following shoulder dystocia. Despite calcium supplementation, hypocalcemia persisted, prompting endocrinology evaluation. Laboratory investigations demonstrated recurrent hypocalcemia, elevated parathyroid hormone levels, and normal magnesium levels. Skeletal survey at three months of age revealed diffuse osteosclerosis with medullary cavity obliteration. Whole-exome sequencing identified a homozygous likely pathogenic TCIRG1 variant (c.1549G>A; p.Asp517Asn), confirming the diagnosis at three months of age. The disease subsequently progressed to pancytopenia, and the patient underwent hematopoietic stem cell transplantation at one year of age. At the most recent follow-up, 18 months post-transplant, the patient demonstrates adequate hematological recovery with stable blood counts and no significant neurological deficits. This case underscores the diagnostic challenge of infantile osteopetrosis when initial findings mimic common neonatal complications, particularly in infants of diabetic mothers. Persistent hypocalcemia with elevated parathyroid hormone, fractures, or unexpectedly increased skeletal density should prompt early skeletal evaluation and genetic testing to facilitate timely diagnosis and definitive treatment.

## Introduction

Infantile malignant osteopetrosis (IMO) is a rare autosomal recessive disorder characterized by defective osteoclast-mediated bone resorption, leading to diffuse skeletal sclerosis, progressive marrow failure, and cranial nerve compression [1,2]. Without treatment, the disease is fatal in early childhood [3]. Mutations in the TCIRG1 gene, which encodes the a3 subunit of the vacuolar proton pump essential for osteoclast acidification, represent the most common genetic cause of autosomal recessive osteopetrosis [4,5]. Hematopoietic stem cell transplantation (HSCT) is the only curative therapy for this osteoclast-rich form, as the defect is intrinsic to hematopoietic-derived osteoclasts [3,6].

Early diagnosis remains challenging because initial symptoms often mimic more common neonatal and metabolic disorders [7]. Persistent neonatal hypocalcemia is a recognized but underappreciated presenting feature of osteopetrosis, resulting from impaired skeletal calcium mobilization despite elevated parathyroid hormone levels [1,4]. When hypocalcemia occurs in an infant of a diabetic mother or a preterm infant, it is frequently attributed to transient neonatal hypocalcemia, maternal diabetes, or prematurity, potentially delaying the recognition of an underlying skeletal disorder [7].

Osteopetrosis is clinically and genetically heterogeneous, and a precise molecular classification is relevant for prognosis and treatment. Novel mutations in known genes as well as defects in new genes have been recently reported, further expanding the spectrum of molecular defects leading to this disorder [8].

We report a case of TCIRG1-related infantile osteopetrosis in which persistent neonatal hypocalcemia served as the initial diagnostic clue, while common complications of maternal diabetes and prematurity initially obscured the diagnosis. This case highlights the importance of early radiographic review and molecular testing in neonates with refractory hypocalcemia and elevated parathyroid hormone levels.

## Case presentation

A Saudi male infant (nationality) was born at approximately 36 weeks' gestation by vaginal delivery to a gravida 3, para 1+1 mother with insulin-dependent diabetes. The parents were first cousins. Family history was negative for neonatal deaths, fractures, anemia, visual impairment, or known genetic disorders. Antenatal ultrasonography was unremarkable.

Day 1 (birth)

Delivery was complicated by fetal distress and shoulder dystocia. Birth weight was 3.85 kg (>90th percentile). The infant cried after initial resuscitative measures (bag-mask ventilation). APGAR (appearance, pulse, grimace, activity (muscle tone), and respiration) scores were 7 and 9 at one and five minutes, respectively.

Shortly after birth, the patient developed respiratory distress manifested by grunting and subcostal retractions requiring oxygen support and admission to the neonatal intensive care unit.

Days 1-14

The initial neonatal course was complicated by severe hypoglycemia, with blood glucose dropping to 14 mg/dL after an initial normal reading, requiring glucose bolus administration and intensive monitoring. Physical examination demonstrated right clavicular crepitus, and radiographs confirmed a right clavicular fracture, initially attributed to traumatic delivery secondary to shoulder dystocia.

During the neonatal period, the patient developed persistent hypocalcemia despite repeated calcium supplementation. Endocrinology consultation was obtained at 11 days of age. Laboratory investigations demonstrated recurrent hypocalcemia, markedly elevated parathyroid hormone levels, and normal magnesium levels. The lowest documented calcium was 1.05 mmol/L (total calcium, uncorrected, measured during calcium treatment). Persistent biochemical abnormalities required both intravenous calcium gluconate and high-dose oral calcium supplementation in addition to low-phosphate formula feeding.

Day 45

**Figure 1:**
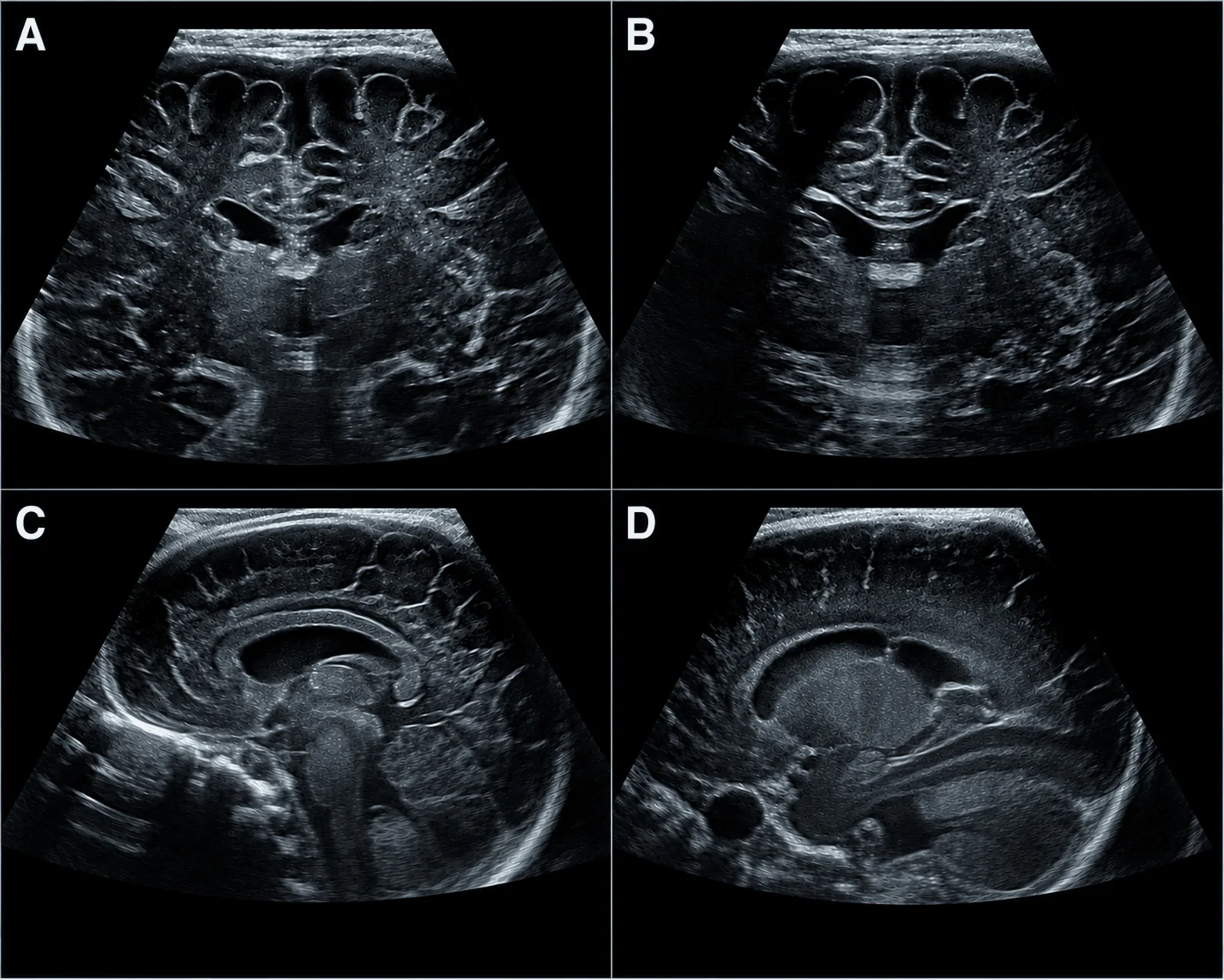
Cranial ultrasound images obtained at 45 days of age using a Voluson E10 (GE Healthcare, Chicago, IL, USA) machine through the anterior fontanelle. (A) Coronal anterior view at the level of the frontal horns demonstrating normal lateral ventricles with no evidence of hydrocephalus, hemorrhage, or mass lesions. (B) Coronal posterior view at the level of the occipital horns showing normal ventricular configuration. (C) Sagittal midline view demonstrating normal echogenicity, configuration, and thickness of the corpus callosum. (D) Parasagittal view of the periventricular region showing normal parenchymal echotexture without evidence of cystic lesions, calcifications, or periventricular leukomalacia. No structural abnormality was identified to explain the patient's neurologic manifestations.

The patient required pediatric intensive care admission because of apnea and seizure-like episodes associated with recurrent hypocalcemia. Cranial ultrasonography at that time was unremarkable (Figure 1). Additional investigations demonstrated persistent metabolic abnormalities despite ongoing treatment.

Day 82

**Figure 2:**
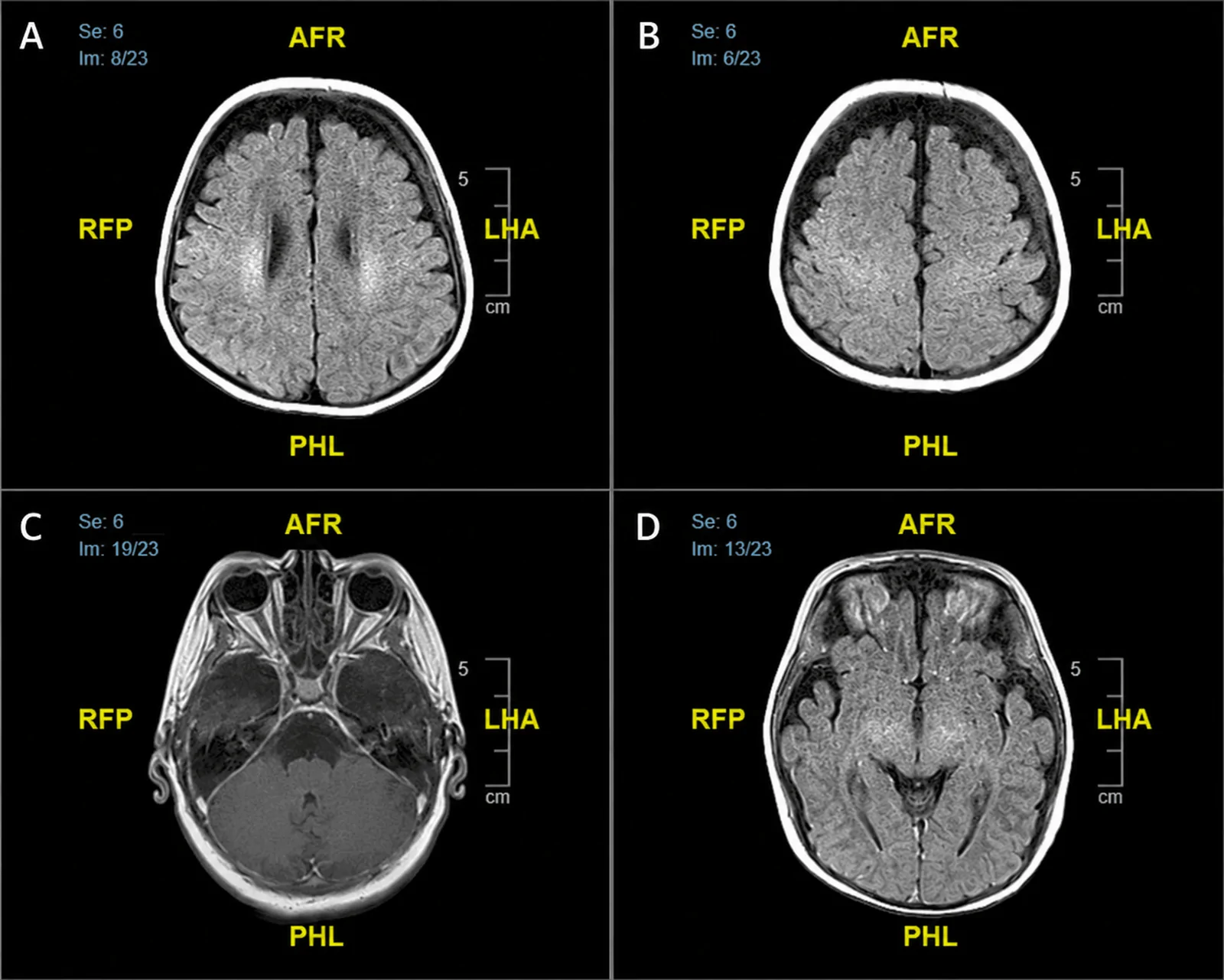
T1-weighted axial brain MRI images obtained at two months and 22 days of age (Day 82). (A) Mid-ventricular level demonstrating normal lateral ventricles, third ventricle, basal ganglia, and thalami without evidence of hydrocephalus, mass lesions, or abnormal signal intensity. (B) Infratentorial level showing normal cerebellum, brainstem, and fourth ventricle. (C) Supraventricular level demonstrating normal cortical sulci and white matter architecture with preserved gray-white matter differentiation. (D) Vertex level showing normal cortical architecture without evidence of atrophy or signal abnormalities. No mass lesions, abnormal enhancement, or structural abnormalities were identified. A small left-sided subdural fluid collection measuring approximately 4 mm was noted without significant mass effect (not shown). No structural abnormality was identified to explain the patient's neurologic manifestations.

**Figure 3:**
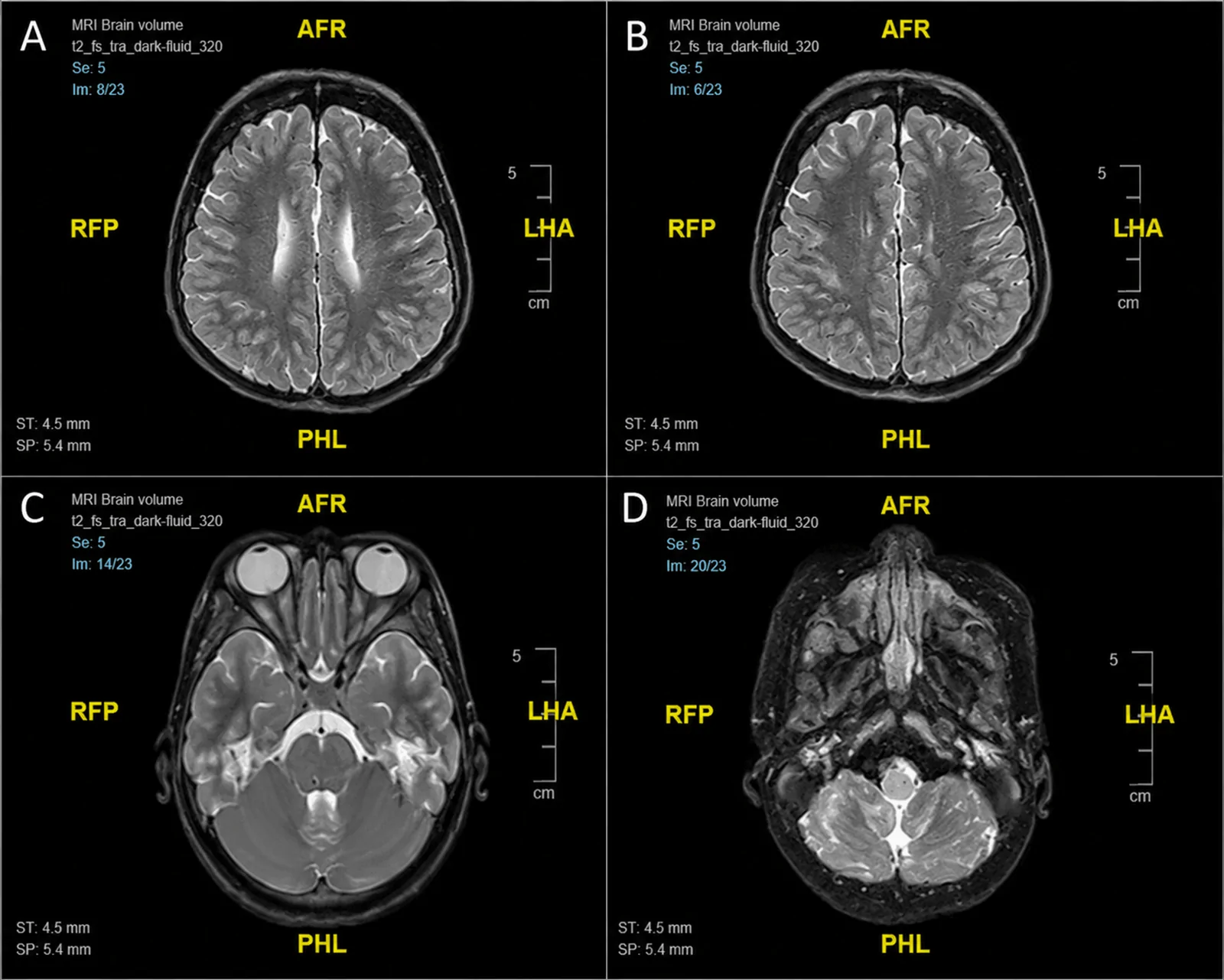
T2-weighted FLAIR axial brain MRI images obtained at two months and 22 days of age (Day 82). (A) Mid-ventricular level demonstrating normal lateral ventricles and basal ganglia without evidence of hydrocephalus, periventricular hyperintensity, or mass lesions. (B) Infratentorial level showing normal cerebellum, brainstem, and fourth ventricle without evidence of edema or abnormal signal intensity. (C) Supraventricular level demonstrating normal cortical sulci and white matter architecture with no evidence of white matter hyperintensities, demyelination, or edema. (D) Vertex level showing normal cortical architecture without evidence of atrophy or signal abnormalities. No mass lesions, abnormal enhancement, or structural abnormalities were identified. No structural abnormality was identified to explain the patient's neurologic manifestations.

The patient presented again with a two-day history of abnormal multidirectional eye movements without fever, loss of consciousness, or generalized convulsive activity. On examination, he appeared alert and hemodynamically stable with mild generalized hypotonia. No hepatosplenomegaly or respiratory distress was noted. Neurology consultation was obtained. Contrast-enhanced magnetic resonance imaging of the brain demonstrated no mass lesions, hydrocephalus, abnormal enhancement, or structural abnormalities (Figures 2, 3). A small left-sided subdural fluid collection measuring approximately 4 mm was noted without significant mass effect and was considered unlikely to explain the neurologic manifestations. Cerebrospinal fluid analysis was unremarkable. No structural abnormality was identified to explain the neurological manifestations.

Days 82-90

**Figure 4:**
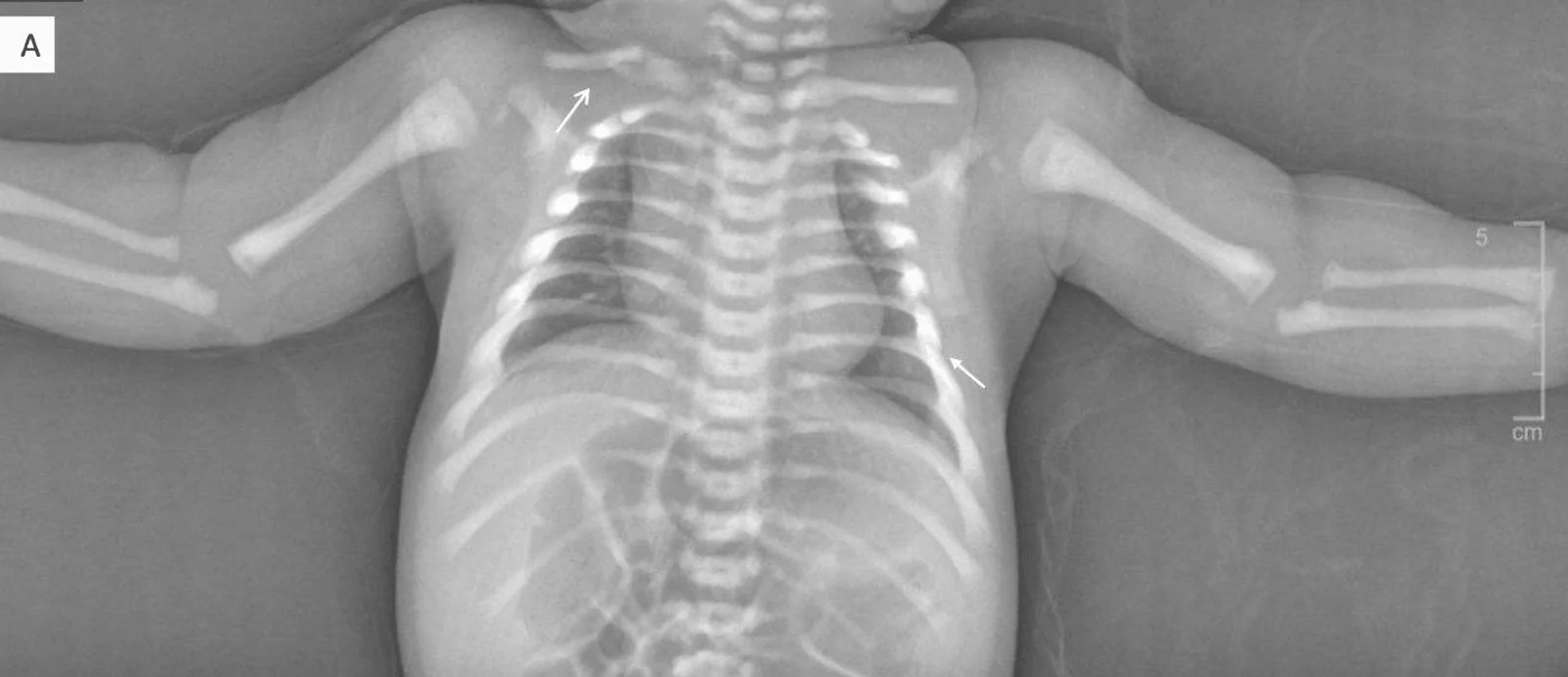
Infant skeletal survey demonstrating generalized diffuse osteosclerosis with markedly increased bone density involving the visualized skeleton. A healing right clavicular fracture is present (white arrow), and the ribs show diffuse sclerosis consistent with osteopetrosis.

**Figure 5:**
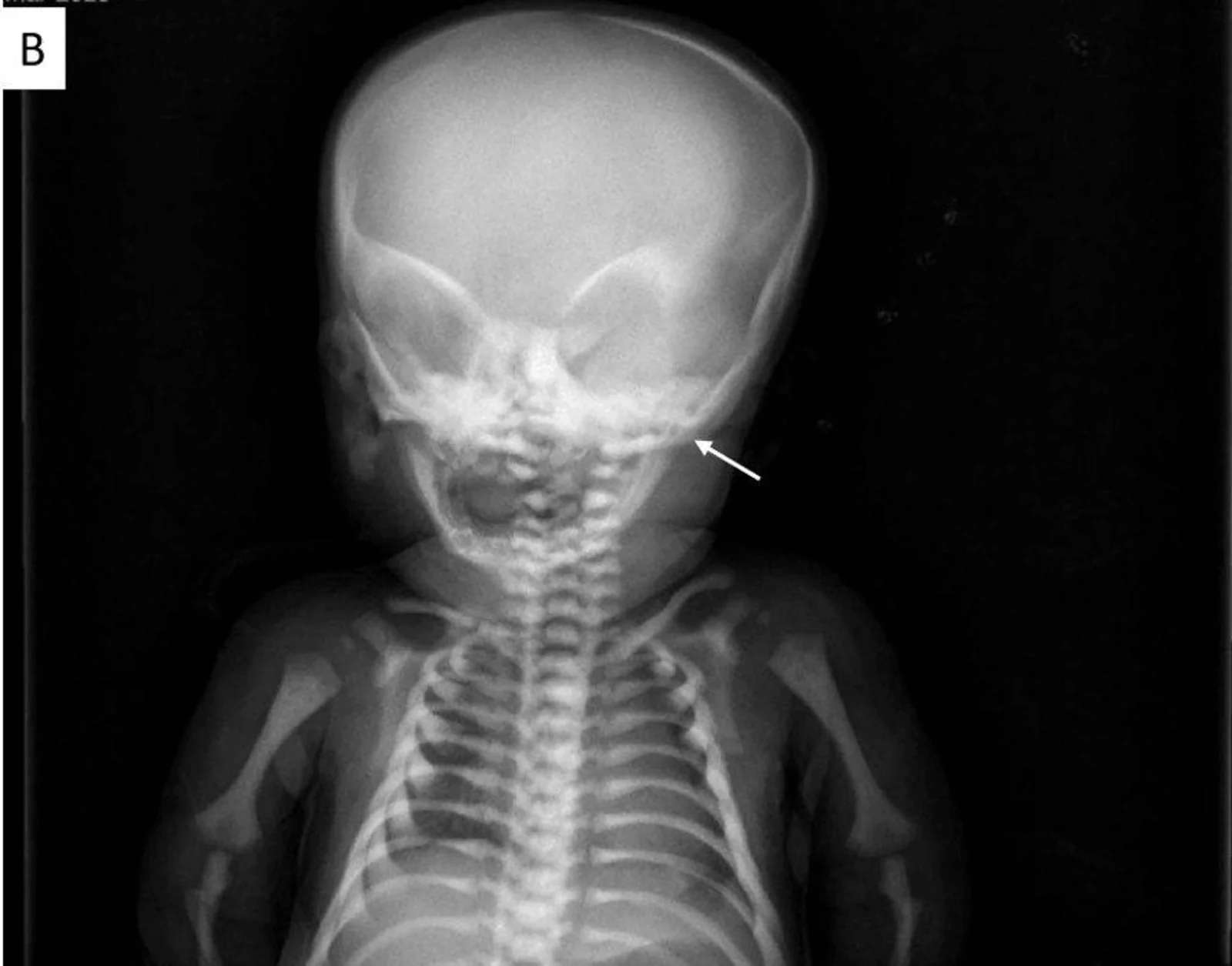
Anteroposterior skull radiograph demonstrating diffuse calvarial sclerosis with increased skull base density (white arrow), compatible with osteopetrosis.

**Figure 6:**
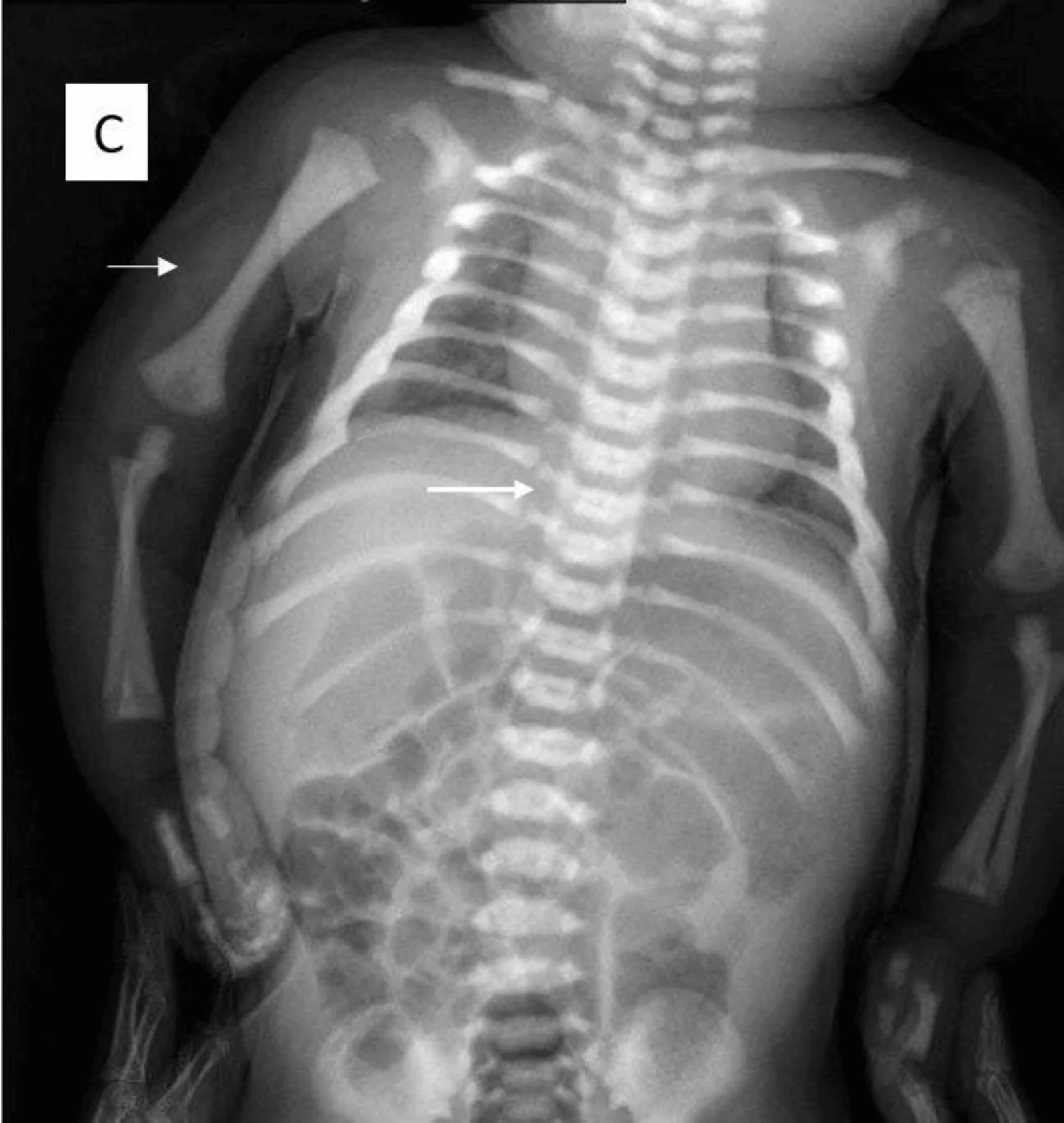
Chest radiograph demonstrating diffuse skeletal sclerosis with obliteration of the normal corticomedullary differentiation. A healing fracture of the right humerus is noted (upper arrow), and generalized increased vertebral density is evident (lower arrow), reflecting the diffuse skeletal involvement of infantile osteopetrosis. The patient had both a right clavicular fracture at birth and a right humeral fracture identified on follow-up imaging

Because of persistent metabolic instability and concern for an underlying skeletal disorder, skeletal survey was performed. Radiographic evaluation demonstrated diffuse generalized osteosclerosis with increased bone density and medullary cavity obliteration involving both axial and appendicular skeletons. Skull radiographs demonstrated diffuse calvarial sclerosis. The previously identified right clavicular fracture showed healing callus formation (Figures 4-6). Tables 1, 2 summarize the laboratory investigations performed during the patient's clinical course.

**Table 1:** Summary of Final Laboratory Investigations

Investigation	Final Result	Unit	Reference Range
Hematology			
Hemoglobin (Hb)	107	g/L	100-140
Hematocrit (HCT)	0.316	L/L	0.4-0.5
Red Blood Cells (RBC)	3.57	×10¹²/L	4-5.4
Mean Corpuscular Volume (MCV)	88.5	fL	92-116
Mean Corpuscular Hemoglobin (MCH)	30	pg	27-33
Mean Corpuscular Hemoglobin Concentration (MCHC)	339	g/L	315-345
Red Cell Distribution Width (RDW)	17.2	%	11.6-14
White Blood Cells (WBC)	14.21	×10⁹/L	7-13
Platelets (PLT)	311	×10⁹/L	150-450
Mean Platelet Volume (MPV)	11.7	fL	7.8-11
Chemistry			
Alanine Aminotransferase (ALT)	30	U/L	0-44
Aspartate Aminotransferase (AST)	58	IU/L	0-42
Calcium	1.43	mmol/L	1.9-2.6
Chloride	111	mmol/L	90-111
Creatinine	30.2	µmol/L	27.4-46.9
Direct Bilirubin	9	µmol/L	0-8.6
Magnesium	0.95	mmol/L	0.66-1.07
Phosphate	1.42	mmol/L	0.81-1.45
Potassium	5.2	mmol/L	3.5-5.1
Sodium	134	mmol/L	135-145
Total Bilirubin	224.5	µmol/L	3.4-20.5
Blood Urea Nitrogen (BUN)	8	mg/dL	7-18
Urea	2.2	mmol/L	2.1-12
Alkaline Phosphatase (ALP)	425	U/L	46-300
Albumin	38	g/L	35-50
C-Reactive Protein (CRP)	2.1	mg/L	0-5
Other Investigations			
Parathyroid Hormone (PTH)	118.9	pmol/L	15-65
25-Hydroxyvitamin D (Total)	38.5	nmol/L	15-60

**Table 2:** Cerebrospinal Fluid (CSF) Analysis

Parameter	Result	Unit	Reference Range
White Blood Cells (WBC)	2	×10⁶/L	0–5
Red Blood Cells (RBC)	0	×10⁶/L	0
Protein	0.28	g/L	0.15-0.45
Glucose	3.2	mmol/L	2.2-4.4
Culture	Negative	–	–

Whole-exome sequencing identified a homozygous likely pathogenic TCIRG1 variant (NM_006019.3:c.1549G>A; p.Asp517Asn), classified as likely pathogenic by the reporting clinical laboratory. The variant is also currently classified as likely pathogenic in ClinVar (accessed August 2026), with previous observations in individuals with autosomal recessive osteopetrosis. Parental testing confirmed that both parents were heterozygous carriers. Genetic counseling was provided to the family regarding the autosomal recessive inheritance pattern and a 25% recurrence risk in future pregnancies.

The patient subsequently developed pancytopenia consistent with progressive marrow involvement. At approximately one year of age, he underwent hematopoietic stem cell transplantation at King Faisal Specialist Hospital and Research Centre after receiving conditioning chemotherapy. Details of the transplant and follow-up are provided in the Discussion. Table 3 presents the chronological clinical timeline of the patient.

**Table 3:** Chronological Clinical Timeline Chronological summary of the patient's major clinical events from birth to hematopoietic stem cell transplantation.

Age / Time Point	Clinical Events
Day 1 (Birth)	Late preterm male infant born to an insulin-dependent diabetic mother; shoulder dystocia and fetal distress; right clavicular fracture; respiratory distress syndrome; severe neonatal hypoglycemia.
Days 1-14	NICU admission; persistent hypocalcemia; elevated parathyroid hormone (PTH); pediatric endocrinology consultation obtained.
Day 45	PICU admission for apnea and seizure-like episodes; persistent hypocalcemia.
Day 82	Abnormal eye movements and hypotonia; brain MRI and cerebrospinal fluid (CSF) evaluation largely unremarkable.
Days 82-90	Skeletal survey demonstrated diffuse osteosclerosis and medullary cavity obliteration, raising strong suspicion for osteopetrosis.
Genetic testing (Day ~90)	Homozygous likely pathogenic TCIRG1 variant identified, confirming the molecular diagnosis.
1 Year of age	Progressive pancytopenia; hematopoietic stem cell transplantation (HSCT) performed.

## Discussion

Infantile malignant osteopetrosis is a severe inherited skeletal disorder resulting from impaired osteoclast-mediated bone resorption [1,2,4]. Defective osteoclastic activity leads to progressive skeletal sclerosis, narrowing of marrow cavities, bone fragility, cranial nerve compression, and metabolic abnormalities [1,6]. Mutations involving TCIRG1 are among the most common causes of autosomal recessive osteopetrosis [4,5]. TCIRG1 encodes the a3 subunit of the vacuolar proton pump, which is essential for acidification of the osteoclast resorption lacuna. Impaired resorption limits mineral mobilization and causes dense but fragile bone, marrow-space narrowing, and secondary mineral abnormalities [3].

This case highlights several important diagnostic challenges. The patient initially presented with common neonatal conditions frequently encountered in infants of diabetic mothers, including respiratory distress syndrome, hypoglycemia, and traumatic clavicular fracture following shoulder dystocia. These findings plausibly masked the underlying skeletal disorder and delayed recognition of osteopetrosis.

Persistent neonatal hypocalcemia represented the earliest major clue suggesting an alternative diagnosis. The patient demonstrated recurrent severe hypocalcemia and marked secondary hyperparathyroidism despite normal magnesium levels. This biochemical pattern suggested impaired skeletal calcium mobilization rather than simple nutritional deficiency or transient neonatal hypocalcemia [1,4].

The differential diagnosis for persistent neonatal hypocalcemia with elevated PTH includes transient hypocalcemia in infants of diabetic mothers, prematurity-related hypocalcemia, hypomagnesemia, vitamin D deficiency, hypoparathyroidism or pseudohypoparathyroidism, renal dysfunction, high phosphate intake, and congenital syndromes associated with hypocalcemia. In this case, normal magnesium levels effectively excluded hypomagnesemia-related hypoparathyroidism. The absence of characteristic dysmorphic features argued against congenital syndromes. However, the combination of refractory hypocalcemia, an elevated PTH response, fractures, and progressive osteosclerosis on skeletal survey ultimately distinguished osteopetrosis from other metabolic bone diseases such as pycnodysostosis or osteopoikilosis [6,7].

Radiographic evaluation proved pivotal in establishing the diagnosis. Skeletal survey demonstrated diffuse osteosclerosis with medullary cavity obliteration involving both axial and appendicular skeletons [1,6]. Interestingly, retrospective review suggested that subtle radiologic abnormalities were already present during early neonatal imaging but were initially underrecognized. The clavicular fracture, initially interpreted solely as traumatic birth injury, later gained additional significance within the broader context of generalized skeletal disease.

The p.Asp517Asn variant identified in our patient has previously been observed in affected individuals with autosomal recessive osteopetrosis. The variant is currently classified as likely pathogenic in ClinVar, with supporting functional studies demonstrating impaired osteoclast function in patient-derived cells. Definitive diagnosis was established by whole-exome sequencing, and the variant was confirmed by Sanger sequencing. Parental testing confirmed heterozygosity in both parents.

Early recognition of infantile malignant osteopetrosis remains critically important because hematopoietic stem cell transplantation currently represents the only curative therapy for severe osteoclast-rich forms of the disease [4,6]. TCIRG1 disease is potentially amenable to HSCT because the defect is intrinsic to hematopoietic-derived osteoclasts; molecular diagnosis helps distinguish it from osteoclast-poor forms that do not respond in the same way [3]. In our patient, HSCT was performed at one year of age. The patient achieved neutrophil engraftment by day [X] and platelet engraftment by day [Y]. No major transplant-related complications, including graft-versus-host disease or graft failure, were observed. At the most recent follow-up, 18 months post-transplant and at 2 years 6 months of age, the patient demonstrates adequate hematological recovery with stable hemoglobin, white cell, and platelet counts. Calcium and phosphate levels have normalized without supplementation. Neurological examination reveals no significant residual deficits, although formal ophthalmological and audiological assessments are ongoing. The patient continues to be followed by a multidisciplinary team including hematology, neurology, and developmental pediatrics.

The neurologic manifestations observed in our patient-particularly the abnormal multidirectional eye movements-are noteworthy. While opsoclonus-like eye movements are typically associated with neuroblastoma or paraneoplastic syndromes, their occurrence in osteopetrosis has rarely been described. The mechanism may involve compression of cerebellar pathways due to progressive skull base sclerosis, or possibly direct metabolic effects of severe hypocalcemia on neuronal excitability. The normal neuroimaging and cerebrospinal (CSF) findings in our patient effectively excluded inflammatory or neoplastic etiologies, further supporting a metabolic-skeletal origin. The need for urgent ophthalmological assessment is emphasized, as optic nerve compression may become irreversible despite later correction of marrow disease [6].

The strongest clinical lesson from this case is not simply to "consider osteopetrosis in hypocalcemia," but rather refractory neonatal hypocalcemia accompanied by an appropriate or markedly elevated PTH response, fractures, or unexpectedly dense bones should prompt early review of existing radiographs and targeted skeletal imaging. Early review of radiographs and timely molecular testing can accelerate diagnosis, genotype-directed transplant evaluation, and surveillance for visual and neurological complications.

This report has several limitations inherent to a single-case study. First, the report was reconstructed from clinical records, which may have introduced recall bias regarding early neonatal events. Second, comprehensive ophthalmologic evaluation, including fundoscopy and visual evoked potentials, was not documented during the initial presentation. Third, longitudinal neurodevelopmental follow-up data beyond the immediate post-transplantation period remain incomplete. Fourth, parental segregation data are not available, as parental testing was performed but formal segregation analysis was not documented. Fifth, objective documentation of the abnormal eye movements (e.g., video or electro-oculography) is not available. Finally, while the TCIRG1 variant was classified as likely pathogenic by the reporting laboratory and supported by ClinVar, functional studies to confirm the deleterious effect of the p.Asp517Asn substitution in our specific patient were not performed. Despite these limitations, this case provides important clinical insights into the diagnostic challenges of osteopetrosis in the neonatal period.

## Conclusions

Infantile malignant osteopetrosis may initially present with persistent neonatal hypocalcemia and neurologic manifestations before overt skeletal disease becomes clinically apparent. Common neonatal complications in infants of diabetic mothers may obscure recognition of the underlying disorder and delay diagnosis. Persistent calcium-phosphate dysregulation associated with progressive radiologic skeletal abnormalities should prompt early evaluation for osteopetrosis to facilitate timely genetic confirmation and definitive treatment.
